# The Impact of Radiation Proctopathy on Secondary-Primary Colorectal Cancer in Patients with Prostate Cancer

**DOI:** 10.1007/s12029-025-01193-0

**Published:** 2025-02-15

**Authors:** Akram I. Ahmad, Zaid Ansari, Tasneem Jamal Al-Din, Ritu Channagiri, Osama Sherjeel Khan, Fernando J. Castro

**Affiliations:** 1 Department of Gastroenterology and Hepatology, Cleveland Clinic Florida, 2950 Cleveland Clinic Blvd., Weston, FL 33331 USA; 2 College of Medicine, Florida Atlantic University Charles E. Schmidt, Boca Raton, FL 33431 USA

**Keywords:** Radiation proctopathy, Proctitis, Prostate cancer, Rectal cancer, Colon cancer

## Abstract

**Purpose:**

We designed this study to evaluate the relationship between radiation proctopathy (RP) and the risk of colon and rectal cancer in prostate cancer patients.

**Methods:**

This is a retrospective cohort study evaluating patients with prostate cancer who received pelvic radiation therapy between January 2004 and January 2024. The study aims to compare the incidence of post-radiation rectal and colon cancer between patients who developed RP and patients who did not. We excluded patients with a previous history of colon cancer, colectomy, or inflammatory bowel disease.

**Results:**

In total, 12,629 met the inclusion criteria, 533 patients were diagnosed with RP, and 12,096 were without. We observed a higher incidence of colorectal cancer (3.75% vs. 0.63%), colon cancer (2.06% vs 0.40%), and rectal cancer (1.69% vs 0.23%) in patients with RP compared to those without PR (*p* < 0.001) during the follow-up period of 81 months for the RP group and 68 months for the non-RP group. PR was associated with colon and rectal cancer with an HR of 4.43 (95% CI, 2.29–8.57; *p* < 0.0001) and 7.27 (95% CI, 3.43–15.43; *p* < 0.0001), respectively.

**Conclusions:**

RP is an independent risk factor for developing rectal and colon cancer after pelvic radiation therapy in patients with prostate cancer.

## Introduction

Pelvic radiation therapy (RT) is a treatment modality for various cancers, including prostate cancer. The rectum and other adjacent organs often fall within the irradiated field resulting in early or late side effects due to the ionizing nature of radiation, which can damage cellular DNA [1]. Radiation proctopathy, or proctitis (PR), is a potential adverse effect of RT, affecting 5–20% of patients [2]. This condition develops from radiation-induced chronic mucosal damage, submucosal scarring, and angiogenesis [2, 3]. Clinically, it presents as urgency, tenesmus, or bleeding after an average latency period of 6 months, although it can manifest much later, and many patients remain asymptomatic [2, 3].

Secondary primary malignancy is a recognized potential side effect of RT. Studies present conflicting results regarding whether pelvic radiation increases the risk of rectal cancer and whether this risk is confined to the irradiated area or affects the entire colon [4–6]. The complexity of this issue arises from the latency period between the initial and secondary malignancies, as well as this population’s increased risk of developing a secondary malignancy [1, 7]. Overall, there is a modest increase in the incidence of rectal cancer following pelvic RT[1, 7].

We hypothesized that the risk of rectal cancer following pelvic RT could be increased by the presence of RP. To test this hypothesis, we designed a study to compare the risk of rectal and colon cancer in patients with RP to those who received radiation but did not develop RP.

## Materials and Methods

We conducted a retrospective, multicenter, longitudinal study across the Cleveland Clinic hospital network. The study received evaluation and approval from the Cleveland Clinic Florida Institutional Review Board.

The study population comprised patients diagnosed with prostate cancer who underwent RT between January 2004 and January 2024. The population was stratified and monitored based on the incidence of post-radiation proctitis into two cohorts: those with RP and those without. The primary outcome measured was the rate of developing rectal adenocarcinoma post-radiation. The secondary outcome was the overall incidence of colorectal cancer.

Data mining techniques were employed to extract patient information from electronic medical records using validated International Classification of Diseases (ICD) codes including K62.7 for RP, C61 for prostate cancer, C18 (C18.0–C18.9) neoplasm of the colon, C19 and C20 for malignant neoplasm of the rectum. Patients included in the study were those who had prostate cancer and subsequently received pelvic RT. Exclusion criteria were patients who underwent colectomy or had a history of colon cancer before RT, as well as those who received radiation for reasons other than prostate cancer. In addition, we excluded patients with inflammatory bowel disease, anal cancer, neoplasia invading the colorectal region, and metastasis.

Further evaluation was performed by manual chart review on the extracted population that developed the primary or secondary outcomes to ensure accurate cohort assignment. Patients were assigned to the RP group if they had proctopathy in a colonoscopy report following radiation, or if colonoscopic rectal images unequivocally showed changes of proctopathy. Patients without definitive reports or clear colonoscopic images were excluded from the study.

Univariate analysis was conducted to examine the relationship between patient characteristics and the presence or absence of RP. The Chi-square test or Fisher’s exact test was used for categorical factors, as appropriate, while the Wilcoxon rank-sum test was applied to continuous factors. Additionally, a multivariable Cox regression model with backward elimination was used to assess differences in the occurrence of colorectal cancer (CRC), colon cancer, and rectal cancer between patients with and without RP. The follow-up period in the model spanned from the time of RT to either the development of the primary outcome or the last available encounter date, whichever occurred first. Kaplan–Meier survival curves were created for these cancer events. A p-value of less than 0.05 was considered statistically significant. All data analyses were performed using SAS version 9.4.

## Results

**Fig. 1:**
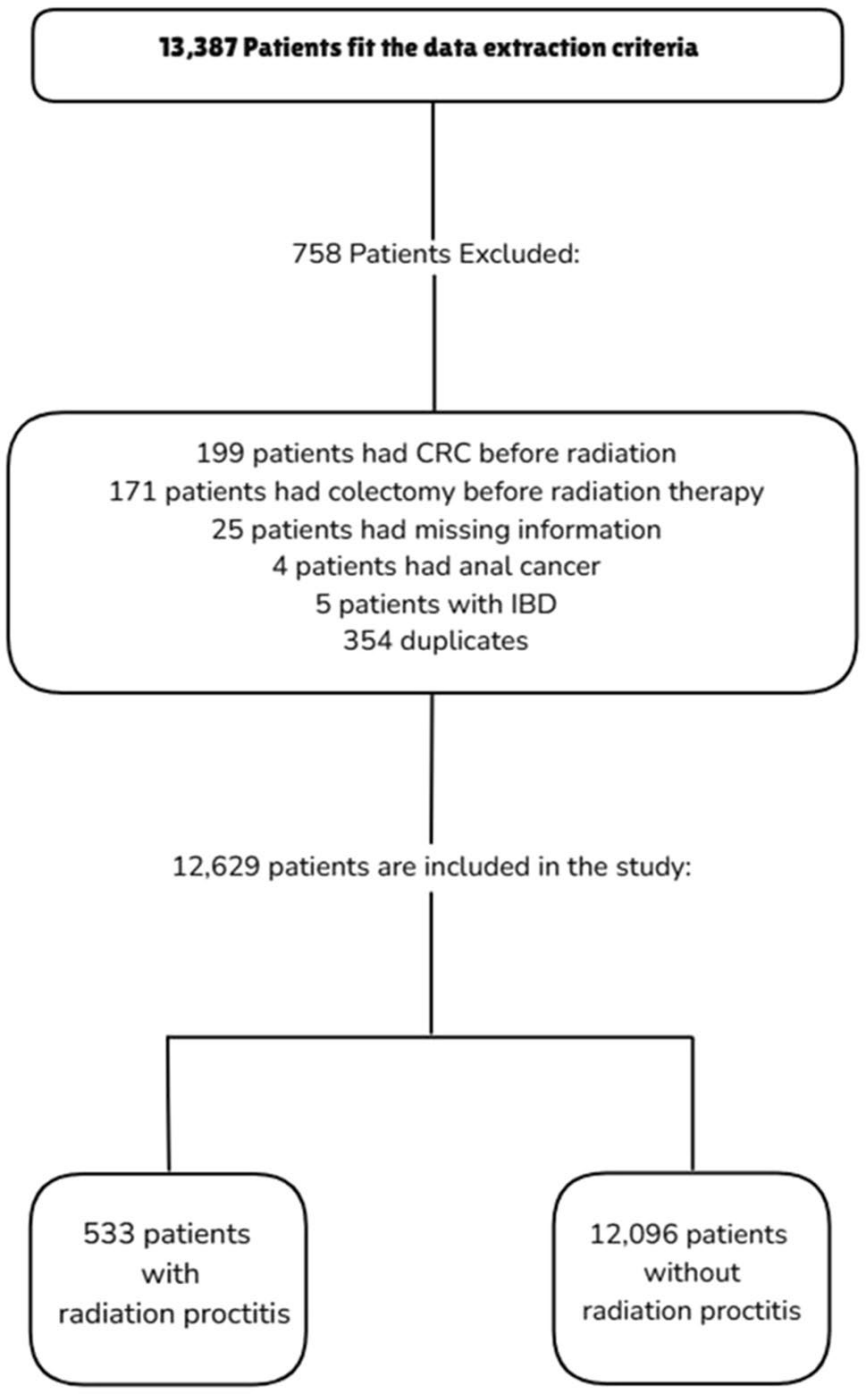
A summary of the study population

A total of 13,387 patients were extracted for analysis based on diagnostic codes. Of those, 12,629 met inclusion criteria and comprised our study population, including 533 patients diagnosed with RP and 12,096 without RP. A summary of the study population is depicted in Fig. 1.

A univariate analysis was performed between the RP and non-RP groups including patients’ age, race, ethnicity, BMI, Charlson Comorbidity Index (CCI), type of radiation, and whether a screening colonoscopy was performed before RT (Table 1). Among these variables, the CCI, radiation type, and prior colonoscopy were significantly unbalanced between the study groups. Specifically, 78.99% of patients who developed RP had a CCI ≥ 5, compared to 63.28% in the non-RP group (*p* < 0.0001). Additionally, a higher percentage of patients in the RP group received external beam radiation therapy (EBRT) (54.9% vs 43.68%) whereas brachytherapy was more common among patients who did not develop RP (51.79% vs 42.40%) (*p* < 0.0001). Moreover, a significantly higher portion of patients who developed RP had undergone colonoscopy before radiation (51.59% vs 40.32%; *p* < 0.0001). The median follow-up period was 81 months for the RP group and 68 months for the non-RP group,

**Table 1:** Patients’ characteristics and outcomes between the groups of proctitis and no-proctitis

Variable	No-proctitis (*N* = 12,096) *n* (%) or median (IRQ)	Proctitis (*N* = 533) *n* (%) or median (IRQ)	*p*-value
Age	69 (63–75)	70 (64–76)	0.1751
Race			0.1267
Black	2115 (17.49)	102 (19.14)	
White	9577 (79.17)	421 (78.99)	
Other/unknown	404 (3.34)	10 (1.88)	
Ethnicity			0.6003
Hispanic	171 (1.41)	9 (1.69)	
BMI			0.1512
BMI < 25	2431 (20.62)	118 (22.74)	
25 ≤ BMI < 30	5015 (42.54)	231 (44.51)	
BMI ≥ 30	4342 (36.84)	170 (32.76)	
Charlson comorbidity index			< 0.0001
(0, 1, 2)	2459 (20.33)	52 (9.76)	
(3, 4)	1983 (16.39)	60 (11.26)	
≥ 5	7654 (63.28)	421 (78.99)	
Radiation type			< 0.0001
External beam radiotherapy	5284 (43.68)	293 (54.97)	
Brachytherapy	6265 (51.79)	226 (42.40)	
Others	547 (4.52)	14 (2.63)	
Colonoscopy done before radiation	48 77 (40.32)	275 (51.59)	< 0.0001
Outcome			
Colorectal cancer (CRC)	76 (0.63)	20 (3.75)	< 0.0001
Colon	48 (0.40)	11 (2.06)	< 0.0001
Rectal	28 (0.23)	9 (1.69)	< 0.0001

Our results revealed an overall incidence of 0.76% for CRC, 0.47% for colon cancer, and 0.29% for rectal cancer. Notably, we observed a significantly higher incidence of CRC (3.75% vs. 0.63%), colon cancer (2.06% vs 0.40%), and rectal cancer (1.69% vs 0.23%) in patients with RP compared to those without RP (*p* < 0.001) (see Table 1).

To assess the independent risk factors for the development of CRC, we performed a multivariable Cox regression analysis (Table 2). This analysis demonstrated that patients with RP had a significantly higher risk of developing CRC compared to those without RP, with a hazard ratio (HR) of 5.20 (95% CI, 3.17–8.55; p < 0.0001). The CCI was also noted to be a significant risk factor, with increasing scores correlating with an increased risk of CRC. Patients with a CCI of 3 or 4 had an HR of 3.36 (95% CI, 1.09–10.30; p < 0.0343), while those with an index of 5 or more had a higher HR of 4.61 (95% CI, 1.68–12.62; p < 0.0030). In contrast, the type of RT showed no significant association with the development of CRC (*p* = 0.1101) and was therefore excluded from the final model using the backward elimination method.

**Table 2:** Multivariable Cox regression analysis for the event of colorectal cancer

Variable	HR	95%CI	*p*-value
Proctitis			
No (ref)	1		
Yes	5.20	(3.17–8.55)	< 0.0001
Charlson comorbidity index			
(0, 1, 2)	1		
(3, 4)	3.36	(1.09–10.30)	0.0343
≥ 5	4.61	(1.68–12.62)	0.0030
Radiation type			
External beam therapy	1		
Brachytherapy	1.37	(0.90–2.08)	0.1467
Others	0.26	(0.04–1.93)	0.1897

When analyzing the risks associated with colon and rectal cancer, RP remained a predictor of both with HR of 4.43 (95% CI, 2.29–8.57; *p* < 0.0001) and 7.27 (95% CI, 3.43–15.43; *p* < 0.0001), respectively (Tables 3 and 4). Additionally, the CCI was found to be a significant risk factor for colon cancer at scores of 5 and higher (HR = 3.91; 95% CI, 1.21–12.57; *p* = 0.0223); however, its influence on rectal cancer was not significant after adjusting for other variables (*p* = 0.1634).

**Table 3:** Multivariable Cox regression analysis for the event of colon cancer

Variable	HR	95%CI	*p*-value
Proctitis			
No (ref)	1		
Yes	4.43	(2.29–8.57)	< 0.0001
Charlson comorbidity index			
(0, 1, 2)	1		
(3, 4)	2.05	(0.51–8.19)	0.3113
≥ 5	3.91	(1.21–12.57)	0.0223
Radiation type			
External beam therapy	1		
Brachytherapy	1.31	(0.77–2.25)	0.3227
Others	0.41	(0.06–3.06)	0.3841

**Table 4:** Multivariable Cox regression analysis for the event of rectal cancer

Variable	HR	95%CI	*p*-value
Proctitis			
No (ref)	1		
Yes	7.27	(3.43–15.43)	< 0.0001
Charlson comorbidity index			
(0, 1, 2)	1		
(3, 4)	7.32	(0.90–59.5)	0.0627
≥ 5	6.70	(0.91–49.39)	0.0619
Radiation type			
External beam therapy	1		
Brachytherapy	1.46	(0.74–2.86)	0.2756
Others	0.00	(NA)	0.9871

**Fig. 2:**
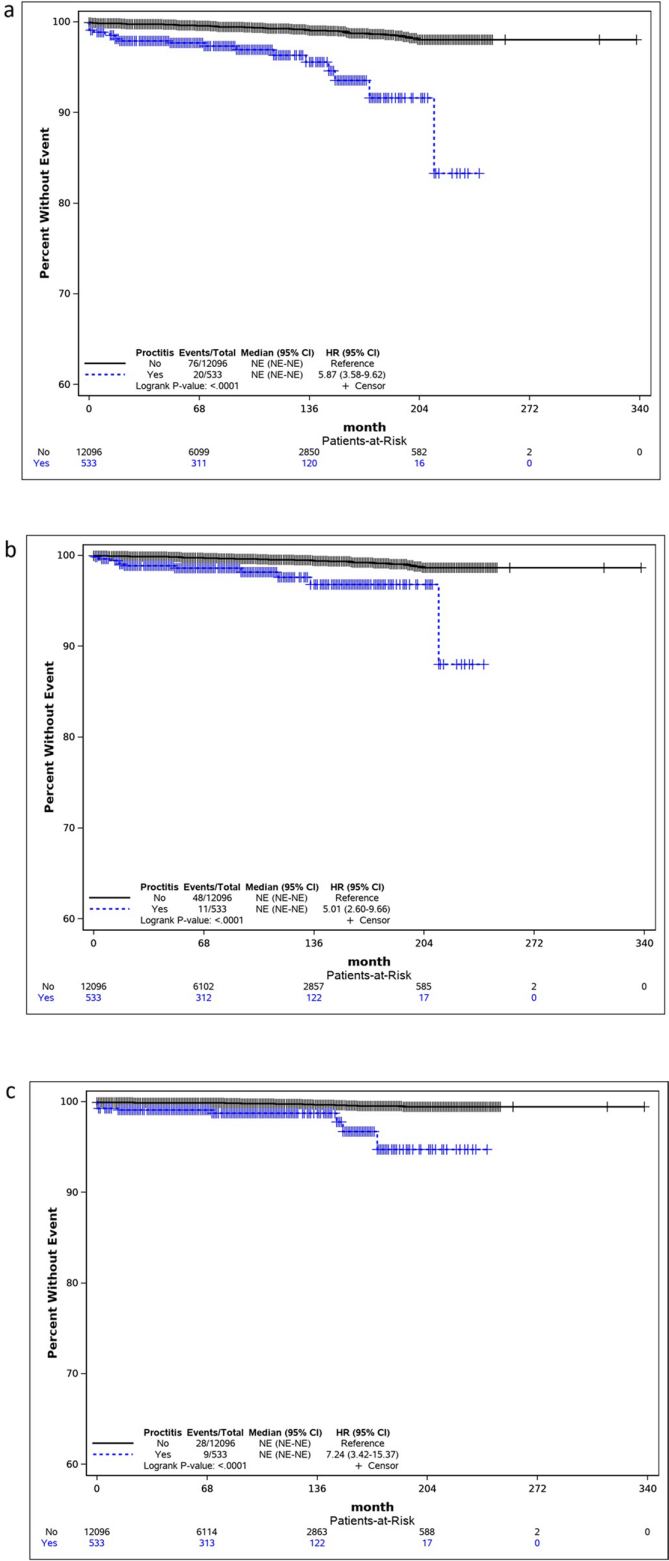
Kaplan–Meier survival curves for the event of **a** colorectal cancer, **b** colon cancer, and **c** rectal cancer

To further analyze these findings, we generated Kaplan–Meier survival curves for CRC, colon cancer, and rectal cancer. The curves consistently showed that patients with RP had a higher probability of developing cancer compared to those without RP. The difference between the survival curves was statistically significant (*p* = 0.0001), as confirmed by the log-rank test. The HR for CRC, colon cancer, and rectal cancer were 5.87 (95% CI, 3.58–9.62), 5.01 (95% CI, 2.60–9.66), and 7.24 (95% CI, 3.42–15.37), respectively (see Fig. 2). The median survival time could not be estimated due to the small number of patients who developed CRC.

## Discussion

The age-adjusted incidence of colon cancer globally is 26.7 per 100,000 individuals [8]. In our cohort, we observed an overall incidence of 0.76% for CRC, 0.47% for colon cancer, and 0.29% for rectal cancer in patients who underwent radiotherapy for primary prostate cancer. These incidences are similar to those reported in previous literature that suggested an increased risk of secondary rectal cancer after pelvic RT. For instance, a meta-analysis reported a pooled incidence of 0.48% for secondary rectal cancer among irradiated patients compared to 0.41% in patients who did not undergo RT as a treatment for prostate cancer (RR 1.36, 95% CI 1.10–1.67) [1]. Similarly, a retrospective cohort study by Baxter et al. [6] found a 0.41% incidence of rectal cancer among irradiated patients in comparison to patients who underwent surgery (0.26%), with an HR of 1.7 (95% CI, 1.4–2.2). Nevertheless, they found no association with colon cancer in potentially irradiated sites such as rectosigmoid, sigmoid, and cecum, as well as in non-irradiated sites [6]. These findings contrast with Rapiti et al. [4] who indicated that irradiated patients had a significantly higher risk of developing colon cancer 5 to 9 years after diagnosis compared to the general population (SIR 4.7, 95% CI 2.0–9.2); however, their data did not show a significant increase of rectal cancer risk in the radiation group. The discrepancy in these results suggests potential factors that may have been underappreciated, such as the role of RP.

While the existing literature has extensively discussed the implications of pelvic radiotherapy on the development of CRC, our study takes a novel approach by specifically isolating and examining the condition of RP as a potential contributor to this elevated cancer risk. We found that RP is associated with an approximately sixfold increase in CRC, fivefold in colon cancer, and sevenfold in rectal cancer. The significant elevation in the risk suggests that RP may be an important factor in the development of cancer. The likely mechanism by which it does so involves a combination of inflammation, fibrosis, and angiogenesis [9], much like the inflammation-dysplasia-carcinoma pathway observed in patients with inflammatory bowel disease (IBD)^7^. This perspective has been largely overlooked in other studies; however, the central question emerging from our findings is whether the observed increased cancer risk is primarily driven by RP itself or by underlying patient characteristics that increase the likelihood of RP.

Our results highlight that individual characteristics may influence the susceptibility to radiation toxicity. A significantly higher percentage of patients with RP had a CCI ≥ 5 compared to those without RP (78.99% vs. 63.28%, *p* < 0.0001). Co-morbidities such as hypertension, diabetes, human immunodeficiency virus infection, and collagen vascular diseases have been discussed in the literature as conditions that increase radiosensitivity [10]. Interestingly, our multivariable Cox regression analysis suggests that the CCI, on its own, can be a significant predictor for CRC and colon cancer at higher scores (HR = 4.61 and 3.91, respectively). To support these results, a population-based study in Australia demonstrated an association between the increase in comorbidity burden (CCI > 2) and the absolute risk of CRC [11]. However, the CCI in our analysis was not associated with rectal cancer after adjusting for other variables. This may imply that the development of rectal cancer in the context of RT may be more directly related to localized tissue damage from RP rather than systemic comorbidities (HR = 7.27, *p* < 0.0001). This is expected as a result of the rectum’s anatomical proximity to the prostate, making it the most affected area during RT.

In addition to characteristic variations, emerging evidence indicates that genetic variants may influence the development of radiation therapy-induced toxicity. For instance, several studies have hypothesized that gene polymorphisms lead to an increased risk of late-onset tissue damage [12–14]. Additionally, specific genes that influence the development of late radiation-induced toxicity have been identified, such as the TANC1 gene found by Fachal et al. [15] in a genome-wide association study. Although these findings provide valuable insights into the underlying mechanisms of radiosensitivity, it is still unclear whether the genetic variability that predisposes individuals to RP also independently influences the development of CRC, or if CRC risk is primarily mediated through the intermediary effect of RP. As genetics continues to be an active field with many unknowns, further research is needed to clarify the interactions between genetic factors, radiation-induced proctitis, and CRC risk.

Moreover, our study adds to the existing evidence stating a higher incidence of gastrointestinal toxicity in patients treated with EBRT compared to brachytherapy [16, 17]. Specifically, RP occurred in 54.97% of EBRT patients versus 42.40% in the brachytherapy group (*p* < 0.0001). However, despite this increased incidence of RP with EBRT, the type of RT did not significantly affect the overall risk of CRC in our final multivariable model. This finding aligns with the work of Wiltink et al. [5], who found that patients who underwent different modalities of RT did not develop more secondary malignancies in the abdominal or pelvic area than nonirradiated patients. This suggests that the risk of CRC may be more dependent on individual factors, such as the development of RP, rather than the specific radiation modality used.

Despite knowing the predictors for chronic RP, its latent presentation remains a challenge for its management. The condition can develop months to years after the completion of RT, often with symptoms that can be minimal or absent, leading to easily overlooked presentations and hence underdiagnosis [18, 19]. A retrospective cohort study investigating the incidence of CRC and RP after prostate radiotherapy using periodic colonoscopy screening reported a 29% cumulative incidence rate of symptomatic RP [20], illustrating the difficulty of solely relying on symptoms for identifying RP. The insidious nature of this condition prompts us to reconsider the potential benefit of implementing a proactive surveillance strategy for patients receiving pelvic RT.

A notable strength of this study lies in its novel focus on the direct relationship between RP and the subsequent risk of CRC, unlike previous studies that have explored the broader implications of RT on CRC. However, despite the significant association and the implications this study holds, it still comes with several limitations. Its retrospective design relies on pre-existing data which may limit our ability to control for all potential confounders. For example, data on patients’ family histories of CRC were not available, as well as the exact radiation dose each patient received, both of which count as factors in assessing CRC risk. Additionally, not all patients in our study underwent a colonoscopy before RT, possibly leading to an underestimation of baseline CRC risk. Although this was relatively balanced between the RP and non-RP groups, it remains a potential source of bias. In addition to that, RP is a rare disease, which leads to a small sample size and low incidence of the primary outcome. Therefore, we propose that future research conduct prospective longitudinal studies with comprehensive data collection to better elucidate the relationship between RP and secondary primary CRC.

In conclusion, this retrospective cohort study provides compelling evidence that RP has a significant association with an increased risk of CRC, colon cancer, and rectal cancer in patients undergoing radiotherapy for prostate cancer during a follow-up period of 81 months for the RP group and 68 months for the non-RP group. Additionally, it identified a high CCI as a potential predictor of CRC and colon cancer, suggesting that the burden of comorbidities may contribute to radiosensitivity and cancer development. These findings pave the way for future studies to clarify the underlying mechanisms and improve cancer prevention and management strategies for prostate cancer patients receiving RT.

## Data Availability

No datasets were generated or analysed during the current study.
